# Deep Vision: An In-Trawl Stereo Camera Makes a Step Forward in Monitoring the Pelagic Community

**DOI:** 10.1371/journal.pone.0112304

**Published:** 2014-11-13

**Authors:** Melanie J. Underwood, Shale Rosen, Arill Engås, Elena Eriksen

**Affiliations:** 1 Institute of Marine Research, Bergen, Norway; 2 Department of Biology, University of Bergen, Bergen, Norway; Aristotle University of Thessaloniki, Greece

## Abstract

Ecosystem surveys are carried out annually in the Barents Sea by Russia and Norway to monitor the spatial distribution of ecosystem components and to study population dynamics. One component of the survey is mapping the upper pelagic zone using a trawl towed at several depths. However, the current technique with a single codend does not provide fine-scale spatial data needed to directly study species overlaps. An in-trawl camera system, Deep Vision, was mounted in front of the codend in order to acquire continuous images of all organisms passing. It was possible to identify and quantify of most young-of-the-year fish (e.g. *Gadus morhua, Boreogadus saida* and *Reinhardtius hippoglossoides*) and zooplankton, including Ctenophora, which are usually damaged in the codend. The system showed potential for measuring the length of small organisms and also recorded the vertical and horizontal positions where individuals were imaged. Young-of-the-year fish were difficult to identify when passing the camera at maximum range and to quantify during high densities. In addition, a large number of fish with damaged opercula were observed passing the Deep Vision camera during heaving; suggesting individuals had become entangled in meshes farther forward in the trawl. This indicates that unknown numbers of fish are probably lost in forward sections of the trawl and that the heaving procedure may influence the number of fish entering the codend, with implications for abundance indices and understanding population dynamics. This study suggests modifications to the Deep Vision and the trawl to increase our understanding of the population dynamics.

## Introduction

Fishery management has shifted focus from a single-species approach towards an ecosystem approach that takes how human interventions and food web linkages affect ecosystems into account [1], [2]. The monitoring programmes used as a basis for fisheries management advice have been forced to adapt to meet the data needs for ecosystem-based management by measuring a wide range of ecosystem components [1], [3]. A number of methods and gears have been employed, ranging from water sampling to plankton nets, pelagic and demersal trawls, grabs and sledges, echo sounders and direct visual observations [4]. Even with modern research vessels, equipment and methods, limitations remain related to gear efficiency and documenting vertical distribution and overlap of organisms. There is thus a need to continue to develop tools and methods in order to overcome these limitations and increase our understanding of population dynamics.

The Norwegian-Russian Barents Sea Ecosystem Survey (BESS) is a comprehensive survey that gathers a wide range of measurements from the physical and biological components of the ecosystem [5]. One task of the BESS is to map the upper pelagic community (including young-of-the-year fish, large krill (Euphausiidae) and jellyfish) with a pelagic trawl to measure abundances and provide biomass indices [6]. However, current survey methods have their limitations, including a lack of spatial distribution data due to all species being collected in a single codend, an inability to identify and quantify less robust species that are destroyed by the codend (e.g. comb jellyfish, Ctenophora) [7] and the difference in the size-catchability performance of the various trawls used [8], [9]. Therefore, it is necessary to develop new tools that can identify and quantify species that are easily damaged and that can sample all species and sizes at the same time.

An in-trawl camera system, Deep Vision (Scantrol AS, Bergen, Norway), has been developed to identify and measure species continuously as they pass inside the trawl [10], [11]. The Deep Vision system has been used for several surveys to identify, quantify and measure the length of large fish such as adult Northeast Arctic cod (*Gadus morhua*), haddock (*Melanogrammus aeglefinus*) and Atlantic mackerel (*Scomber scombrus*) along the trawl track. Time-references for each image can be matched up with data such as geographic position and acoustic backscatter information collected by the vessel's echo sounder [12]. The stereo images can be processed to calculate the size of passing objects with high accuracy (less than 5% error; [10]) as well as the spatial distribution along the trawl path. Our goal was to evaluate if the current system can be used to identify, quantify and measure small and fragile organisms, and how it can be improved to meet the goal of collecting data to increase our understanding of population dynamics.

## Materials and Methods

### Ethics Statement

BESS is conducted as part of the Norwegian and Russian obligations under the international law for the monitoring of environmental changes and the management of living marine resources, approved by the Joint Norwegian-Russian Fisheries Commission and the Norwegian Ministry of Fisheries and Coastal Affairs. The collection of image data was carried out as part of the BESS. No endangered or protected species were encountered during the field studies, and fish acquired by trawling were immediately killed when they came onboard. Collection of the image data had no additional impact on the welfare of the organisms. The standard biological sampling procedures are routine work at sea, approved by the Institute of Marine Research Institutional Animal Care and Use Committee.

### Study area and trawling procedure

**Figure 1:**
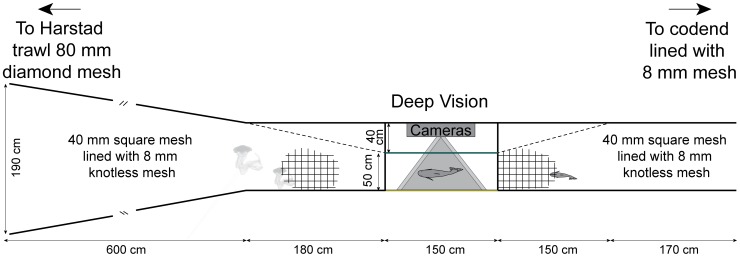
Schematic representations of the Deep Vision frame and trawl section.

Deep Vision observations were carried out during standard sampling hauls on the BESS inside the Isfjord and Billefjord areas at Svalbard in August 2012 with RV “Johan Hjort”. The trawl was towed at three depths (with the headline at 0, 20 and 40 m) for 0.5 nautical miles each, at a speed over ground of 3 knots [6]. The survey uses a pelagic standard sampling trawl for young-of-the-year fish [Harstad sampling trawl; 8]. The four panel trawl consists of seven sections with mesh sizes ranging from 200 mm in the front of the trawl to 8 mm in the codend [13]. The Deep Vision section was attached to the trawl between the extension and the codend (Fig. 1). The trawl dimensions (i.e. vertical opening of the net mouth, wing spread and depth of the headline) were measured with acoustic trawl instrumentations (SCANMAR AS, Åsgårdstrand, Norway). The catch was measured using the BESS standard biological sampling procedure [14]. Images from Deep Vision were analysed post-cruise.

### Deep Vision camera system

The Deep Vision camera system consists of two 1.4 megapixel digital colour cameras fitted with 4.8 mm focal length lenses and arranged in a parallel stereo orientation. The cameras are connected to a PC for control and data storage and placed inside a subsea housing rated to 200 bar pressure. Illumination is provided by two external light emitting diode (LED) strobes which generate 38 400 lumens. With such a high level of artificial illumination, there is very little difference between images collected at the surface and at depth. Batteries provide power for up to eight hours of operation. A pressure sensor continuously collects system depth data (5 m resolution) and images are time-stamped to match them with external sensor data.

Five images per second were collected, starting before the trawl was set out and ending when it came back onboard the vessel, saving a continuous time-referenced record of all objects that passed through the trawl during the shooting, trawling, and heaving phases. The system and its measurement accuracy are described in detail by Rosen *et al* [10] and Rosen and Holst [12]. In this study, the system was operated in autonomous mode without cable connection to the vessel.

The camera housing, lights, and battery were mounted inside a 90 cm high ×90 cm wide ×150 cm long neutrally buoyant frame made of 10 mm-thick high-density polyethylene (HDPE). The frame was placed in a 12.5 m-long four-panel net section between the extension and the codend (Fig. 1). The whole Deep Vision section was lined with 8 mm mesh similar to the codend in order to guide all the catch in front of the camera. The leading end of the net section was 190 cm in height and width, corresponding to a minimum sampling cross-section of 3.6 m^2^ for individual organisms that were not herded by the larger meshes in the trawl. Tapering and lead nets immediately in front of the HDPE frame guided the catch through a passage within the field of view of both cameras at a range of 27 to 73 cm (field of view  = 41 and 102 cm width, respectively). The camera side of the passage was made of 10 mm-thick transparent polycarbonate while the back wall, roof, and floor were opaque to retain light and provide contrast with the edges of the passing fish. The area surrounding the camera was also constructed of opaque white material in order to provide diffused, even illumination.

### Image Analysis

Both images and biological samples were collected on eight hauls. Two hauls were selected for post-cruise image analysis to evaluate if the current system can be used to identify, quantify and measure small and fragile organisms. In order to provide the most diverse samples of fish and zooplankton, one haul with the greatest number of commercially important finfish (haul 04; 11∶56 UTC) and one with the greatest number of zooplankton with finfish present (haul 06; 3∶29 UTC) were selected. All images collected in the course of these hauls were reviewed in order to identify and quantify the finfish and zooplankton that passed the Deep Vision camera. The depth at which each individual was imaged was determined by matching the image timestamp with the time-referenced depth recorded from the pressure sensor in the camera housing. Data from haul 04 were analysed to compare spatial distributions between species, while data from haul 06 were analysed to compare length measurements made by the Deep Vision system and those of the actual catch.

**Figure 2:**
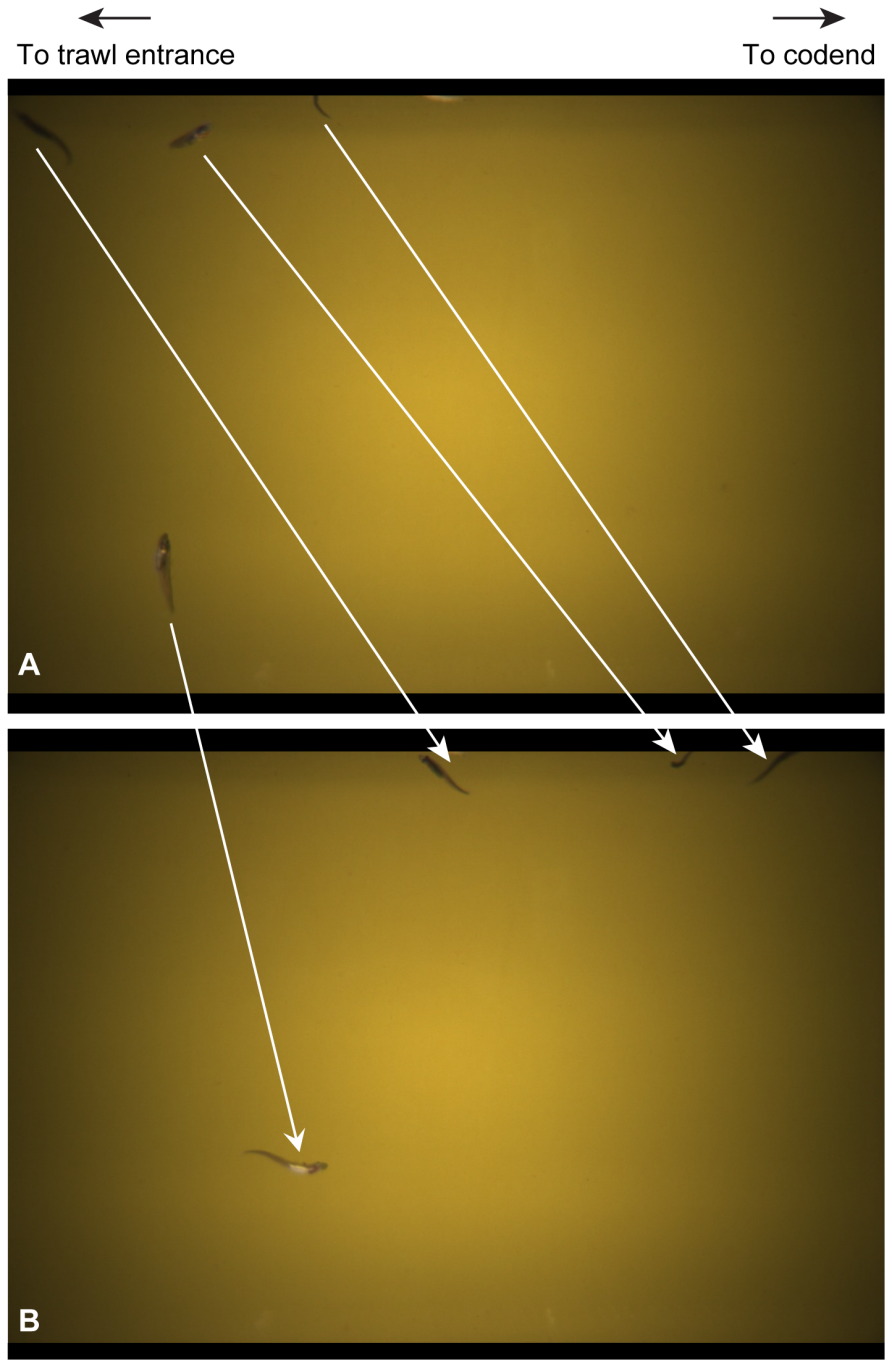
Example of tracking fish through sequential images. (A) Four polar cod (*Boreogadus saida*) enter the Deep Vision chamber and (B) move towards to the codend in the next image, taken 200 ms later. The white arrows show the movement by each individual.

A total of 21 030 images from haul 04 and 19 714 images from haul 06 were analysed in two ways. First, all finfish and lion's mane jellyfish (*Cyanea capillata*) were counted, in accordance with the BESS catch sampling protocol. Most individuals were imaged several times as they passed through the 41–102 cm field of view (generally taking 3 images or 600 ms to pass). In order to prevent double-counting, individuals were tracked across images (Fig. 2) and counted in the image when they first entered the field of view. Individuals that exited the field of view from the direction of the trawl entrance were subtracted from counts and added again when they re-entered the field of view. Individuals that could not be identified were recorded as ‘unidentified’. Second, all other zooplankton were quantified by sampling the first image of each 30-second interval and counting the number of individuals present (a total of 140 images from haul 04 and 124 images from haul 06). This method was used because it was impossible to track small zooplankton between images when densities were high (hundreds of individuals in a single image). Since there was a 30-second interval between images, it is unlikely that any individuals were double-counted. To estimate the total count of zooplankton in each haul, each sub-sample representing the number of passages in 600 ms was multiplied by 50 to estimate total number of individuals passing in each 30-second interval.

Lengths of polar cod (*Boreogadus saida*), shorthorn sculpin (*Myoxocephalus scorpius*) and Northeast Arctic cod were measured using the Deep Vision software [10], and compared with the codend catch data to test the possibility of using the Deep Vision for measuring the lengths of small organisms. Measurements were limited to species with visible caudal fins (i.e. Greenland halibut were not used), since the catch data were recorded as total length (from the snout to the end of the caudal fin) and individuals presenting both snout and caudal fin to the camera. Since the catches of polar cod were large (4588 individuals), lengths were measured from a random sub-sample, consistent with the BESS protocol. The first Deep Vision image of every 15-second interval was analysed and lengths were calculated for all polar cod orientated such that they could be measured. The differences between the length measurements from the catch data and the image-estimated lengths of polar cod were analysed by one-way ANOVA.

## Results

### Trawl geometry and performance

**Figure 3:**
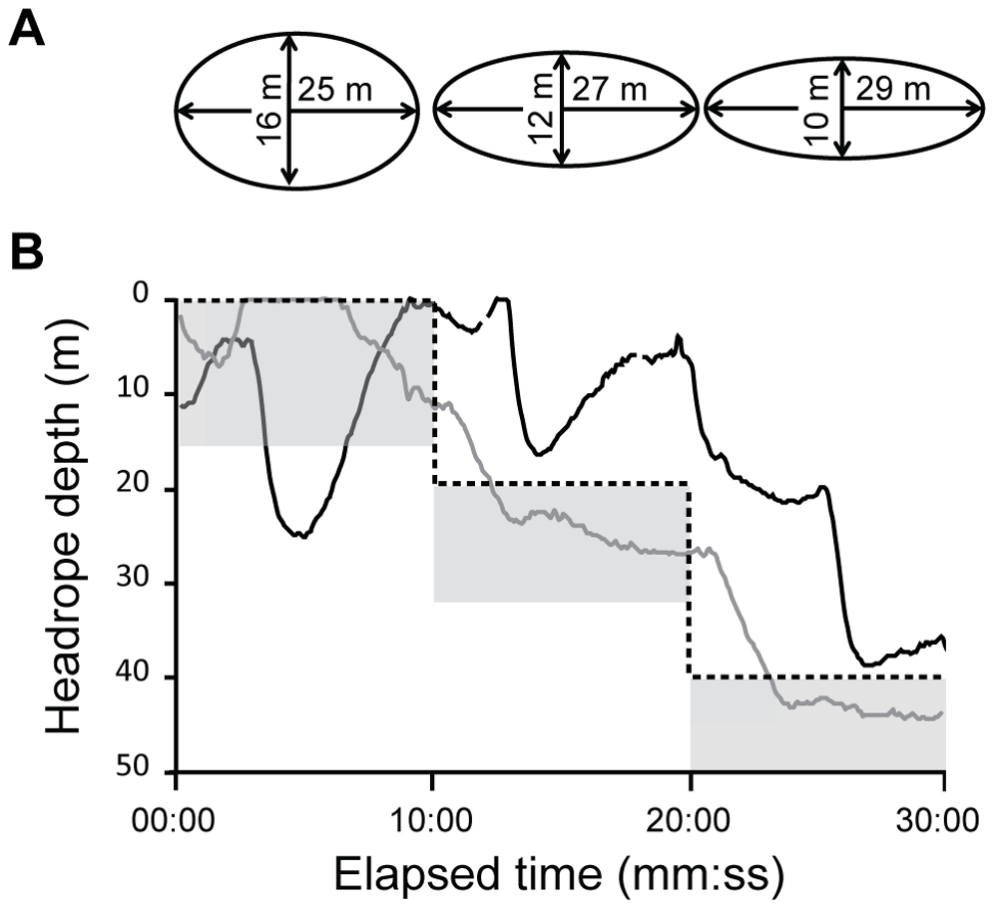
Schematic representation of the (A) net mouth geometry and (B) headline depth. The black line indicates the headline depth for haul 04, while the grey line indicates the headline depth for haul 06. The dashed line shows the stepwise protocol for BESS. The shaded areas under the dashed line indicate the height of the trawl mouth opening and the depths surveyed.

The depth sensor showed that the path of the trawl in the water column deviated from the survey protocol (Fig. 3). Measurements of the trawl geometry also showed that the vertical opening and wing spread changed with depth. The vertical opening of the trawl diminished from approximately 16 to 10 m with the headline at 0 m and 40 m respectively, while the corresponding wingspread measurements increased from approximately 25 to 29 m.

### Deep Vision images

We were able to identify and quantify from the Deep Vision images eleven species of juvenile finfish, including six species used for species index analysis, and five zooplankton species (including comb jellyfish; Table 1). However, some organisms could not be identified to species level and some were present in the catch but not observed in the images. We were unable to consistently discriminate between redfish (*Sebastes* spp.) and polar cod, and counts of polar cod may therefore include redfish. This was also the case with capelin (*Mallotus villosus*) and shannies (Stichaeidae), and counts of shannies may therefore include capelin. The problem was greatest when individuals passed the camera at the maximum range of 73 cm. Four haddock were found in the catch but not identified in the images. Total counts of small zooplankton per haul were generally overestimated compared to the catch data when extrapolated from the 30-second sub-sample.

**Table 1:** List of species and families identified and quantified in the Deep Vision images and in the catch data.

	Counts from the Deep Vision and Catch data for each haul			
	Haul 04		Haul 06	
Species/family	Images	Catch data	Images	Catch data
Greenland halibut *Reinhardtius hippoglossoides*	50	51	6	5
Capelin *Mallotus villosus* a	NA	1184	NA	1
Norwegian spring spawning herring *Clupea harengus*	1	4	0	1
Northeast Arctic cod *Gadus morhua*	18	22	1	1
Redfishes *Sebastes* spp.^a^	NA	551	NA	0
Polar cod *Boreogadus saida*	19431	29268	2114	4588
Shorthorn sculpin *Myoxocephalus scorpius*	274	248	2	2
Shanny family Stichaeidae	1734	1680	3332	5797
Lumpfish Cyclopteridae	4	4	0	0
Snailfish family Liparidae	119	193	32	12
Atlantic poacher *Leptagonus decagonus*	1	1	12	4
Lion's mane jellyfish *Cyanea capillata*	44	38	144	81
Haddock *Melanogrammus aeglefinus*	0	4	0	0
Unidentified	25		3	
Krill *Thysanoessa* spp.^b^	2450	330	19000	9020
Comb jellyfish Ctenophora^b^	30000	NA	38250	NA
Amphipods *Themisto* spp.^b^	2200	4067	8700	7447
Sea butterfly Thecosomata^b^	600	413	950	111

Species and families unable to be quantified are marked as NA.

a Redfishes were difficult to distinguish from polar cod, and capelin were difficult to distinguish from the shanny family. Both were not counted.

b Small individual zooplankton were unable to be tracked between images and therefore were sub-sampled every 30 s. Counts from Deep Vision are estimated from the sub-sample.

**Figure 4:**
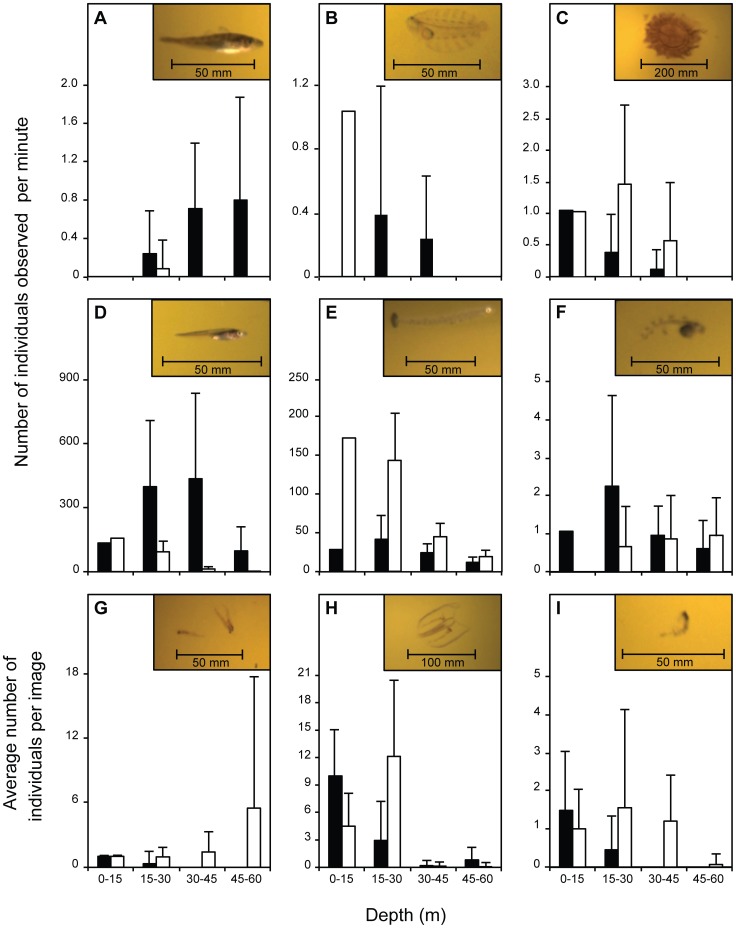
Species caught at various depths by two image-sampling methods during the haul. (A–F) sample all images and show the number of individuals observed per minute for each species/family and (G–I) are sub-sampled (first image of each 30-second interval) and indicate the average number of individuals per image, at four different depths. The dark column bars indicate haul 04 while haul 06 is shown by white column bars. The species/families are (A) Northeast Arctic cod (*Gadus morhua*; Haul 04, *n* = 15; Haul 06, *n* = 1), (B) Greenland halibut (*Reinhardtius hippoglossoides*; Haul 04, *n* = 10; Haul 06, *n* = 1), (C) lion's mane jellyfish (*Cyanea capillata*; Haul 04, *n* = 10; Haul 06, *n* = 25), (D) polar cod (*Boreogadus saida*; Haul 04, *n* = 12633; Haul 06, *n* = 1443), (E) shanny family (Stichaeidae; Haul 04, *n* = 1162; Haul 06, *n* = 2531), (F) snailfish family (Liparidae; Haul 04, *n* = 58; Haul 06, *n* = 23), (G) krill (*Thysanoessa* spp.; Haul 04, *n* = 15; Haul 06, *n* = 130), (H) comb jellyfish (Ctenophora; Haul 04, *n* = 151; Haul 06, *n* = 344), and (I) amphipods (*Themisto* spp.; Haul 04, *n* = 25; Haul 06, *n* = 66). The error bars indicate the upper standard deviation. The trawl spent just one minute in the 0–15 m depth, therefore no standard deviation was calculated for (A–F).

**Figure 5:**
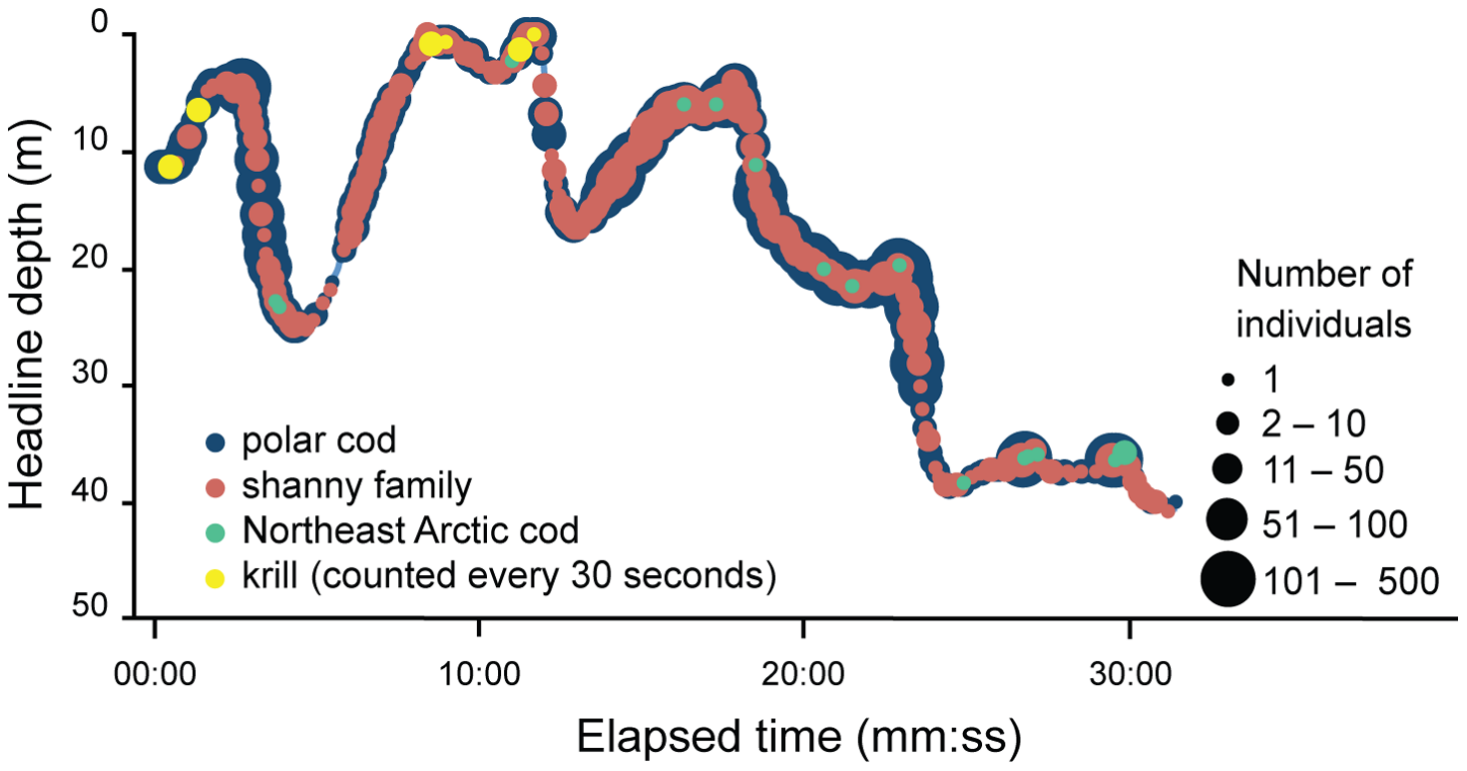
Species distribution and abundance throughout haul 04. Species include polar cod (*Boreogadus saida*), shanny family (Stichaeidae), Northeast Arctic cod (*Gadus morhua*) and krill (*Thysanoessa* spp.).

**Figure 6:**
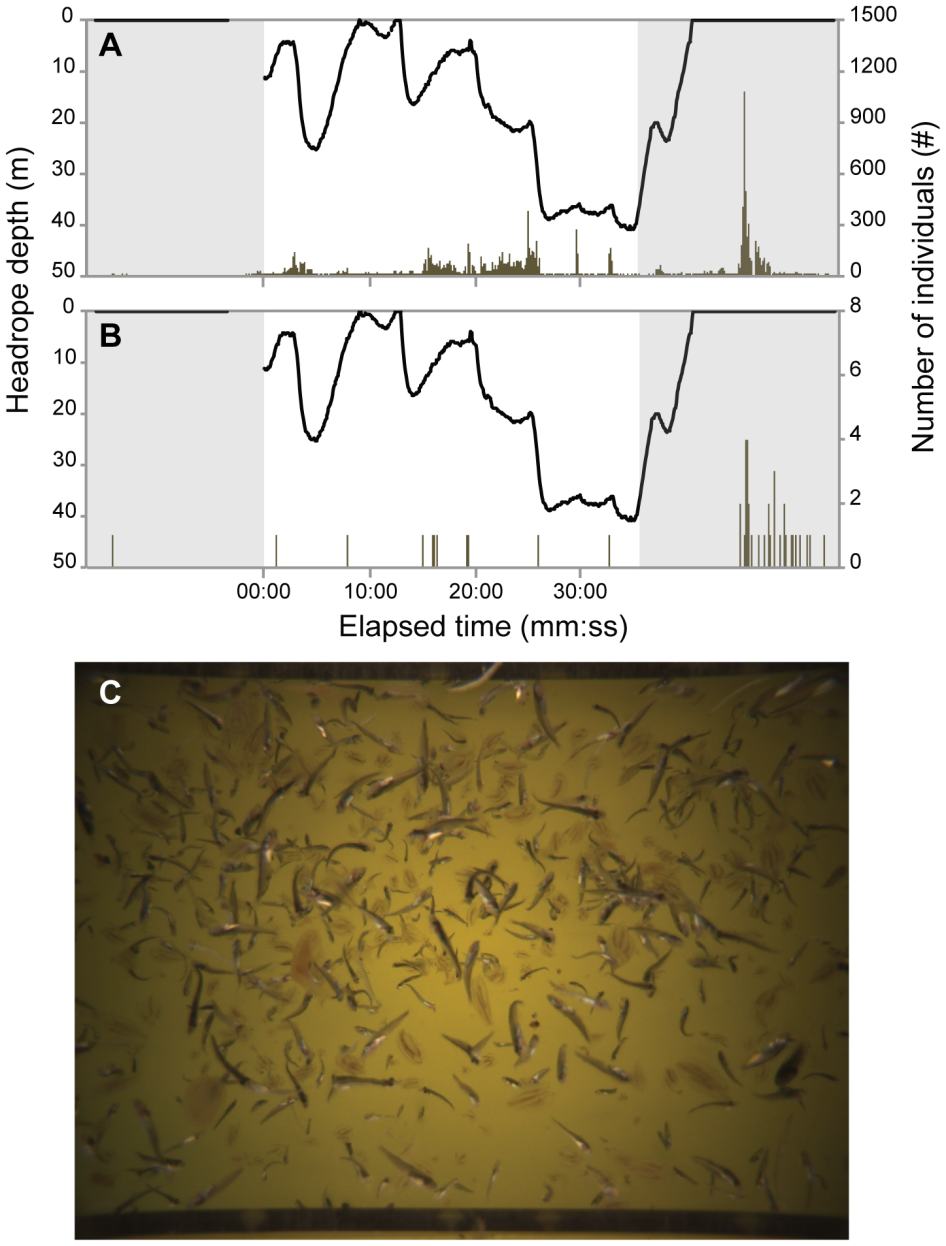
Density of two species during all phases of trawling, haul 04. Species include (A) Polar cod (*Boreogadus saida*) and (B) Greenland halibut (*Reinhardtius hippoglossoides*). The grey area on the left represents shooting and the grey area on the right signifies heaving. Lower image (C) is an example of an image during heaving.

Individuals were observed at all the depth layers during the standard 30-minute towing time, with most species increasing in number down to 30 m and then decreasing at greater depths (Fig. 4). However, numbers of Northeast Arctic cod and krill continued to increase as the depth increased. Species were observed to enter the codend in patches and with other species during haul 04 (Fig. 5). Polar cod and shannies were observed together at all depths throughout haul 04. A large number of polar cod and Greenland halibut were observed to pass the camera when the trawl was at the surface during heaving with up to 35% of polar cod and 80% of Greenland halibut passing outside of the designated 30-minute trawling time (Fig. 6). During heaving, when the trawl was already at the surface, more than 280 polar cod per second passed the Deep Vision system, with individuals moving ahead (towards the trawl entrance) in patches for short periods of time before re-entering the field of view. This made it difficult to quantify young-of-the-year fish during heaving when high densities and turbulent flow were observed. Counts from the images may therefore be underestimates. Individuals of all species were observed entering the codend in groups during heaving (at the surface) and some were seen to have damaged opercula.

**Figure 7:**
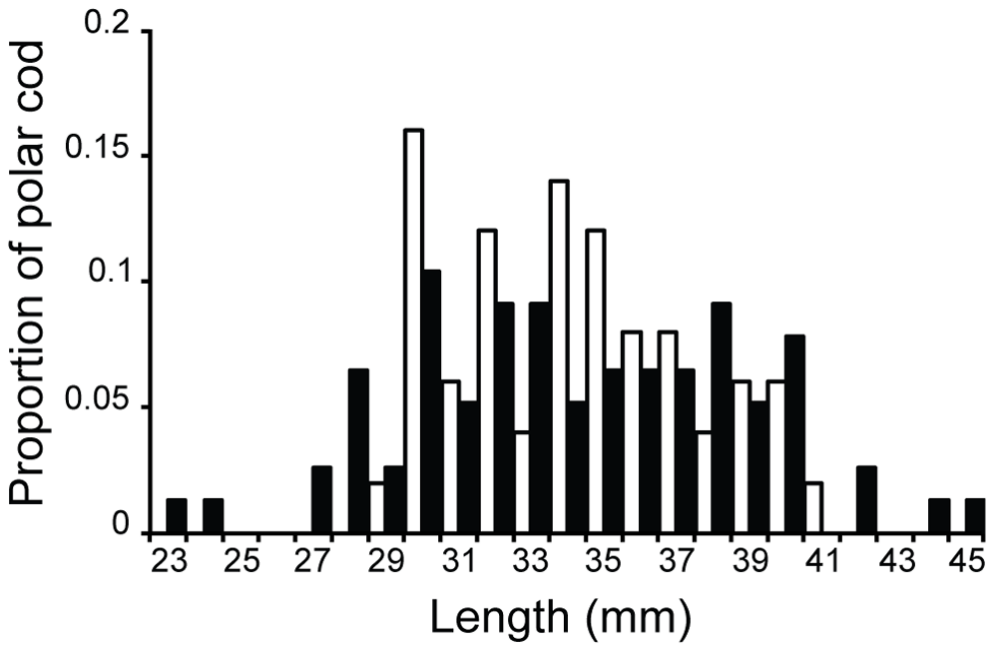
Length comparison of polar cod (*Boreogadus saida*) measured by two different methods. The dark column bars indicate measurements from the Deep Vision data (*n* = 77, mean  = 34 mm), while the catch data measurements are indicated by white column bars (*n* = 50, mean  = 34 mm).

Fifty polar cod were measured from the catch and 77 out of 115 individuals were measured from the images for length comparisons (the remaining 38 were not imaged in orientations where lengths could be estimated). Average lengths of polar cod were not significantly different in the images and catch data (mean length 34 mm for each method; F = 0.034, *p* = 0.85; Fig. 7). The single Northeast Arctic cod was measured at 53 mm in both the Deep Vision image and the catch data. Only the larger of the two shorthorn sculpin was passed in an orientation where it could be measured. Its length was calculated to be 43 mm in the Deep Vision image compared to 45 mm in the catch data (4.4% difference).

## Discussion

The pelagic community is a vital component of the Barents Sea ecosystem, providing important links between lower and higher trophic levels [15], [16]. Pelagic fish species such as capelin, herring (*Clupea harengus*) and polar cod make up the bulk of the total biomass and are mainly plankton-feeders (consuming primarily Euphausiacea and Amphipoda). The Barents Sea is also a nursery area for several commercially and ecologically important fish stocks, with young-of-the-year distributed in the upper water column during the summer and autumn. The pelagic community is surveyed by acoustics, plankton nets and trawls during BESS. The use of several sampling methods with different efficiencies, sampling volumes and deployment times makes it difficult to observe species overlaps, which are essential for studies of small scale processes, such as interspecies interactions (competition, mutualism, protocooperation, predation etc.), which are important to understand the population dynamics [17]. There is thus a need for new tools that can measure several components of the ecosystem simultaneously and can provide greater spatial resolution than single-codend trawling. The Deep Vision system has shown promising results for larger species [11] and this study is the first step towards verifying whether the current Deep Vision can be used to identify, quantify and measure small organisms continuously as they pass inside the trawl.

### Benefits and limitations of the Deep Vision system

The current Deep Vision was able to identify, and quantify most passing small organisms, as well as species damaged in the codend (e.g. Ctenophora). The system showed potential for measuring the length of small organisms and also recorded the vertical and horizontal positions where individuals were imaged. Limitations became apparent with the current system and modifications should be made and tested.

The Deep Vision system was designed with a camera resolution and field of view suitable for observations of large opaque fish (40–70 cm), and some limitations were seen with observing smaller and transparent individuals within the Deep Vision chamber. For instance, it was difficult to identify species with similar body shape, particularly when they passed the camera at maximum range (73 cm). This was particularly noticeable with shannies as they could not be identified below family level and were difficult to distinguish from capelin. Similarly, redfish and polar cod were also difficult to tell apart. This problem may be mitigated by increasing the image resolution either by reducing the maximum range at which objects pass the camera or by using higher resolution cameras. Furthermore, adjustments to the lighting setup to make transparent organisms more visible in the images should be tested.

High densities of fish and turbulence inside the Deep Vision chamber made it difficult to track individuals during heaving. This was especially evident for polar cod and shannies, which showed the greatest differences between catch and image counts. Reducing the sampling period to during the designated haul (i.e. not to include setting out and heaving) would make tracking individuals more precise and increase the accuracy of quantifying fish using the Deep Vision system. Mismatch between the counts of small zooplankton from the images and codend catch may be the result of the image sub-sampling interval coinciding with the passage of high density patches or incomplete emptying of the codend when it was brought back onboard.

This study indicated the Deep Vision holds promise for measuring the length of small organisms. For polar cod which were oriented such that they could be measured with the Deep Vision, the mean length was the same as the catch data. For the Northeast Arctic cod and sculpin, the individual lengths differed by less than 5%, a similar result as for larger individuals by Rosen *et al* [10]. Due to the large number of individuals orientated poorly during high densities (i.e. parts of individuals hidden by other organisms), it became apparent that the current method would not be efficient for length studies of small organisms. Therefore, a more in-depth study on the length verification of small organisms using a modified trawl with the Deep Vision is recommended. It would be beneficial to establish a partial body to total length ratio for each species, as seen for larger fish in Rosen *et al* [10] to reduce the number of fish unable to be measured due to body position. In addition, a multi-sampling codend [18], [19] can be used to collect sub-samples over shorter time periods for verifying the accuracy of the Deep Vision results. Finally, manually analysing 20,000 images per haul required a substantial amount of time and an automated system would be a major advance. Software for automating tasks such as eliminating empty images, object counting, measurement and species identification is currently under development.

### Survey and ecological implications

Trawls are known to be species- and size- selective [20]. In order to use the Deep Vision with trawls for ecological studies, an understanding of the trawl efficiency and the rate at which organisms pass through the trawl is needed.

High numbers of fish were observed to pass the Deep Vision showing signs of damaged opercula. The damaged opercula indicate that the fish had been caught in the meshes before passing the camera, and it is assumed that a portion of the meshed fish were flushed out of the trawl rather than moving back past the camera and into the codend. This was supported by the observation of fish caught in the middle-sized meshes ahead of the Deep Vision section when the trawl was brought on deck. However, it was not possible to estimate the meshing rates for different species or sizes. This may have implications for ecological studies due to the delay of when meshed individuals entered the trawl and when they were imaged in the Deep Vision, as well as the loss of individuals during towing. For the small individuals observed in this study that did not contact the mesh, it is assumed that due to their poor swimming capacity, the passage rate back into the trawl is similar to the speed of the trawl through water [21].

Previously meshed fish were primary observed passing through the Deep Vision during heaving which may have been due to the way that the trawl was handled and/or sea state (i.e. netting alternately slack and taut from stopping/starting heaving and wave activity). The proportion of fish entering the codend verses escaping may therefore have differed from haul to haul, which may have influenced species composition, number of individuals and length distribution of the catch, which in turn may have influenced the abundance index. The proportion of fish entering the codend rather than escaping during heaving could be reduced by using the information from the Deep Vision system to quantify species only during the designated trawling time and modifying the trawl construction to prevent individuals from becoming meshed.

The two hauls we analysed did not follow the standard protocol and the time spent at each depth range was unevenly distributed. The change in trawl geometry with depth meant that the full upper 60 m of the water column was not sampled, which may have produced a change in the catch efficiency of the trawl with depth. However, since each image captured by the Deep Vision has a depth associated with it, the catch data could be weighted by the amount of time spent at each depth range, reducing the importance of equal depth sampling.

The Deep Vision has the potential to be a valuable addition to the tools available for monitoring the upper pelagic community. Further development of this system to improve the analysis of images of small organisms and current survey methods could provide an increased understanding of population dynamics. In addition to further development of Deep Vision system itself, we need to design and test a trawl that performs consistently at all depths and that prevents organisms from becoming meshed before they enter the codend. One way forward might be to construct the front of the trawl with large square meshes to prevent herding and meshing of small organisms, combined with trawl doors that fully spread the trawl at the surface. Farther back in the trawl, the large square meshes would be lined with overlapping sections of small mesh netting to prevent meshing and escapes. Similar techniques are used in the commercial krill fishery in the Antarctic sea and were successfully tested during the 2013 BESS.
